# Word2vec convolutional neural networks for classification of news articles and tweets

**DOI:** 10.1371/journal.pone.0220976

**Published:** 2019-08-22

**Authors:** Beakcheol Jang, Inhwan Kim, Jong Wook Kim

**Affiliations:** Department of computer science, Sangmyung University, Seoul, South Korea; Politechnika Krakowska im Tadeusza Kosciuszki, POLAND

## Abstract

Big web data from sources including online news and Twitter are good resources for investigating deep learning. However, collected news articles and tweets almost certainly contain data unnecessary for learning, and this disturbs accurate learning. This paper explores the performance of word2vec Convolutional Neural Networks (CNNs) to classify news articles and tweets into related and unrelated ones. Using two word embedding algorithms of word2vec, Continuous Bag-of-Word (CBOW) and Skip-gram, we constructed CNN with the CBOW model and CNN with the Skip-gram model. We measured the classification accuracy of CNN with CBOW, CNN with Skip-gram, and CNN without word2vec models for real news articles and tweets. The experimental results indicated that word2vec significantly improved the accuracy of the classification model. The accuracy of the CBOW model was higher and more stable when compared to that of the Skip-gram model. The CBOW model exhibited better performance on news articles, and the Skip-gram model exhibited better performance on tweets. Specifically, CNN with word2vec models was more effective on news articles when compared to that on tweets because news articles are typically more uniform when compared to tweets.

## Introduction

Deep learning is a field of machine learning that has attracted significant attention following the release of AlphaGo, which was developed by Google in 2016. Recently, various open source deep learning libraries such as Google’s TensorFlow [1], Berkeley’s Caffe [2], University of Montreal’s Theano [3], and SkyMind’s Deeplearning4J [4] were developed, thereby making it easier for individuals to develop deep learning programs[1–4]. They are used in various studies for various purposes such as data classification [5], behavior recognition [6], and event detection [7–9]. High-performance GPUs aid in the resurrection of deep learning by reducing complex matrix calculation times used in deep learning [10, 11]. Additionally, vast amounts of big web data are generated through the Internet, and a large amount of data and tag information generated by online news and Twitter correspond to good learning materials for deep learning systems [12–14].

Evidently, news articles and tweets are appropriate for deep learning that requires large amounts of data to be effective because they are updated in real time and constantly accumulated. However, they typically contain unnecessary text, such as advertisements and alternative uses of words, which may interfere with accurate learning [15]. Thus, it is necessary to filter out unnecessary articles and tweets from the collected articles and tweets although it is not possible to manually classify unnecessary articles and tweets in a large number of articles and tweets.

Word2vec is a word embedding technique that was proposed by Mikolov et al. [16, 17] in 2013 for word expression including the meaning and context of words in a document and includes two learning algorithms, namely continuous bag-of-word (CBOW) and skip-gram algorithms. The similarity among words calculated via the cosine similarity of word vectors in word2vec includes the meaning of words in the document and this exceeds that of other word embedding techniques [18], and thus, several studies such as emotion analysis, emotion classification, and event detection use word2vec [19–21]. A convolutional neural network (CNN) is an artificial neural network that is frequently used in various fields such as image classification, face recognition, and natural language processing [22–24]. In the field of natural language processing, CNN exhibits good performance as a neural network for classification [25].

Given the importance and utilization of news articles and tweets, the word embedding capability of word2vec and the classification ability of CNN for deep learning, they were examined in several studies [25–27]. A previous study [25] proved that a pre-trained word vector is an important factor in sentence classification by comparing a random word vector with a pre-trained word vector. An extant study[28] compared the performance of CBOW and Skip-gram, two learning algorithms of word2vec. A previous study [29] indicated that dual word embeddings performed better when compared to a single word embedding in document ranking. Another study[30] examined the effect of character-level CNN on text classification. Researchers [8] conducted a study to detect events via tokenizing a given document and predicting event triggering tokens. A previous study [7] proposed Multi-Group Norm Constraint CNN (MGNC-CNN) via multiple word embedding to improve the sentence classification performance of CNN. An extant study[9] proposed a neural network model that was independent of the language via extracting features of sentence structures as opposed to words. However, to the best of the authors’ knowledge, extant studies [7–9, 25, 28–30] did not explore word2vec’s CNN classification effects on various parameters, such as the learning frequency and the training volume, in news articles and tweets.

In the present study, we explore the performance of word2vec [16, 17] CNNs [31] to classify necessary and unnecessary documents in news articles and tweets. Previous studies in [18, 28] compared the performances of the Skip-gram and CBOW for Wikipedia and medical journals, and studies in [25, 32] compared the performance of the CNN classification model for various data such as tweets, movie reviews, and customer reviews. However, any of previous studies did not compare the performance of Skip-gram with CBOW for news articles and tweets and did not consider the impact of training data size and the number of epochs in training the CNN classification model. In this paper, we evaluate the performance of the word2vec CNN classification model as a function of the data size and the number of epochs. To evaluate the classification accuracy of CNN with word2vec models, we performed extensive experiments on large sets of real tweets and news articles. The experimental results indicated that the use of word2vec that learns semantic relations among words significantly improved the accuracy of the classification model. The accuracy of the CBOW model is higher and more stable when compared to the Skip-gram model. The CBOW model exhibited better performance when used on news articles and the Skip-gram algorithm exhibited better performance when used on tweets. Additionally, CNN with word2vec models were more effective for news articles when compared to tweets because news articles typically exhibit more uniform formats when compared to tweets. It is expected that the results of the study will clearly reveal how the use of word embedding models affects news articles and tweets classification via CNN.

The structure of this paper is as follows. First, we present background knowledge and related research on word2vec and CNN. Second, we describe the data used and the proposed classification model. Third, we show the experimental results. Finally, we describe the discussion, conclusions, and future work.

## Background knowledge and related works

In this section, we describe word2vec and CNN algorithms and summarize the related works.

### Background knowledge

#### Word2vec

**Fig 1:**
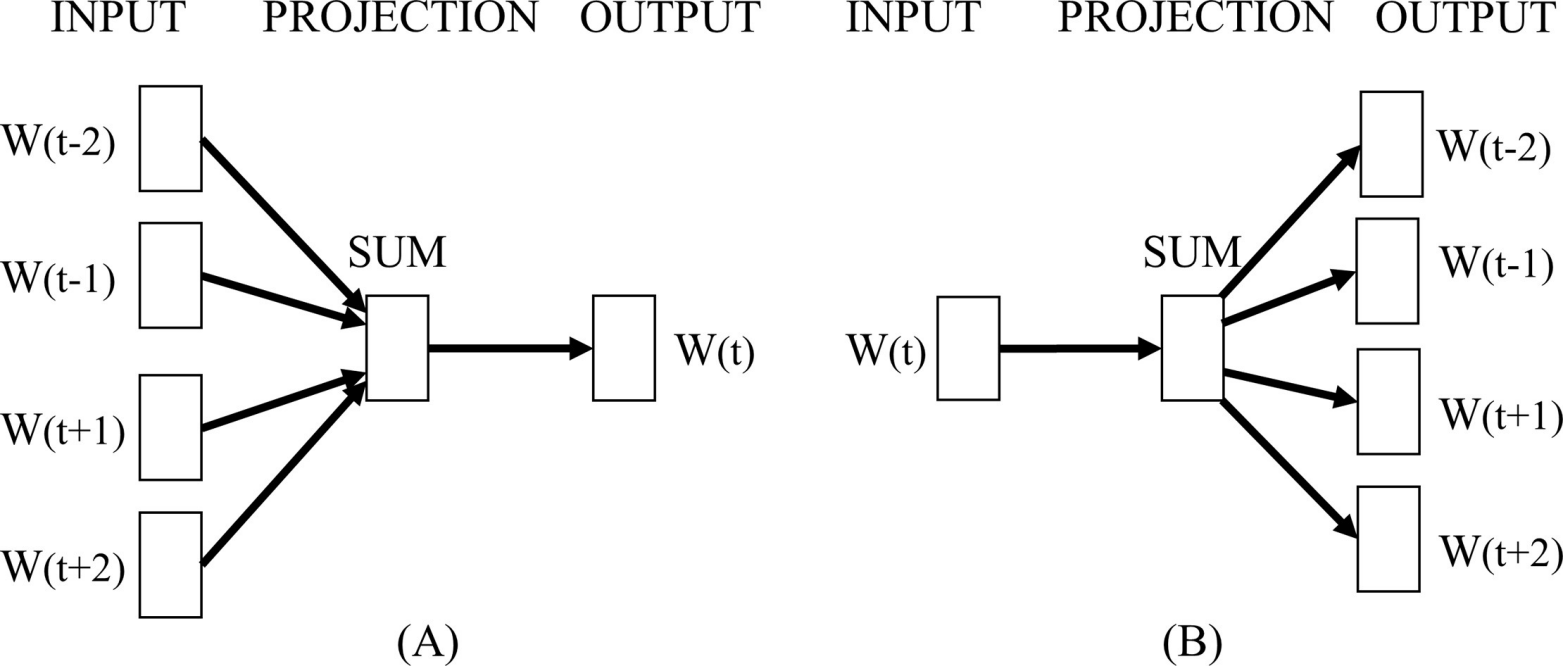
Model architecture of (A) CBOW and (B) Skip-gram.

Word2vec understands and vectorizes the meaning of words in a document based on the hypothesis that words with similar meanings in a given context exhibit close distances [33]. Fig 1 shows the model architectures of CBOW and Skip-gram, learning algorithms of word2vec proposed by Mikolov. Both the learning algorithms exhibit Input, Projection, and Output layers although their output derivation processes are different. The input layer receives W_*n*_ = {W_(t−2)_,W_(t−1)_,…,W_(t+1)_,W_(t+2)_} as arguments, where *W*_*n*_ denotes words. The projection layer corresponds to an array of multidimensional vectors and stores the sum of several vectors. The output layer corresponds to the layer that outputs the results of the vectors from the projection layer. Specifically, CBOW is similar to the feedforward Neural Network Language Model (NNLM) [34] and predicts the output word from other near word vectors. The basic principle of CBOW involves predicting when a certain word appears via analyzing neighboring words. The projection layer of CBOW projects all words at the same position, and thus, the vectors of all words maintain an average and share the positions of all words. The structure of CBOW exhibits the advantage of uniformly organizing the information distributed in the data set. Conversely, the Skip-gram exhibits a structure for predicting vectors of other words from one word. The basic principle of Skip-gram involves predicting other words that appear around a certain word. The projection layer of the Skip-gram predicts neighboring words around the word inserted into the input layer. The structure of the Skip-gram exhibits the advantage of vectorizing when new words appear. Based on the study by Mikolov, CBOW is faster and better suited when compared to Skip-gram when the data size is large, and Skip-gram exhibits a better performance when compared to CBOW while learning new words. However, other studies that compare the performance of CBOW and Skip-gram state that the performance of Skip-gram exceeds that of CBOW [18, 28].

#### CNN

**Fig 2:**
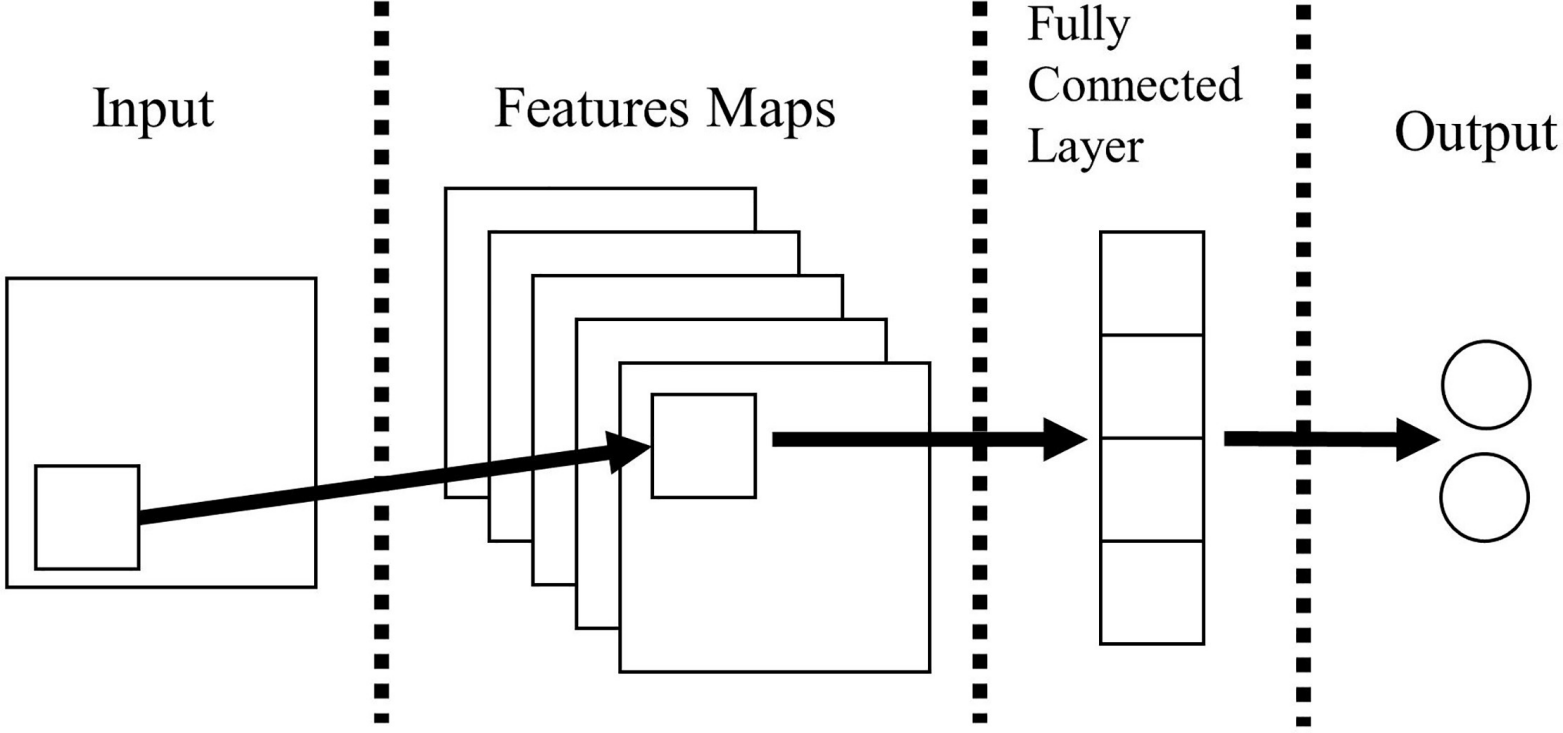
The general model of the CNN.

Fig 2 presents the general model of CNN [31]. CNN is a neural network that is useful for extracting and classifying features because it can pass values to the next layer without losing spatial information. These features of CNN are suitable for utilizing spatial information such as semantic similarity between words in a sentence. CNN consists of input, multiple hidden, and output layers. The layers consist of feature maps and a fully connected layer with convolutional layers and pooling layers. The convolutional layer and the pooling layer extract the characteristics of the input values and map the extracted values to the feature map. In this process, the characteristics of the sentences can be extracted through the semantic similarity between the words constituting the sentence, and then the fully connected layer has a classification value from features extracted for classification. The process of CNN is as follows. Within the input layer, parsed data is passed to the feature maps. The data is stored at a specific location in the convolutional layer of the feature maps. The convolutional layer performs a convolutional operation on the data and maps it to a pooling layer. The data performs a max pooling operation before it is mapped to the pooling layer. The max pooling involves extracting the largest value of the previous layer's results. Subsequently, CNN creates a fully connected layer that combines all convolutional and pooling layers. The fully connected layer finally outputs the result to an output layer.

### Related works

#### Word2vec

Lai et al. semantically and syntactically evaluated the performances of word embedding learning algorithms including NNLM [34], Log-Bilinear Language Model [35], Collobert and Westor (C & W) [36], and word2vec [18]. In their evaluation, they confirmed that the performance of word2vec exceeds that of other algorithms. They used Wikipedia and New York Times articles as learning data sets for word embedding and used semantic and syntactic performance evaluation methods such as WordSim353 set [37], TOEFL set [38], analogy task [16] using CNN, and POS tagging. The performance evaluation indicated that the performance of Skip-gram exceeded that of CBOW in sentence classification via CNN although it did not examine a performance comparison with other factors such as iteration or the effect of training size on CNN performance.

Chiu et al. compared the performance of CBOW and Skip-gram, which are two learning algorithms of word2vec [28]. They used biomedical-related data as learning data sets. The performance of both algorithms was also evaluated using UMNSRS-Sim and UMNSRS-Rel [39], which exhibit biomedical-related word pairs including the similarity of 566 and 587 word pairs. They evaluated the performance of the algorithms via comparing the similarity extracted from each learning algorithm. They indicated that the performance of Skip-gram exceeded that of CBOW. This was in contrast to Mikolov’s claim that CBOW exhibited better performance for news data. The aforementioned difference indicated that the performance of algorithms is dependent on the type of learning data, and this implies that different results may be obtained while using Twitter as learning data.

Ling et al. mentioned that the lack of sensitivity of word2vec to the order of words is useful for semantic expression although this causes it to exhibit a suboptimal performance while solving grammatical problems [40]. To solve this issue, they proposed the Structured Skip-gram that modified the Skip-gram and Continuous Window (CWindow) that modified the CBOW. The proposed two algorithms predicted word orders and words in the output layer. Wikipedia and Twitter were used for the learning data, and POS (Part-Of-Speech) Tagging was used for the evaluation. When compared to existing CBOW and Skip-gram, Skip-gram exhibited better performance and Structured Skip-gram exhibited better performance when compared to CWindow and Structured Skip-gram. Although the study used Twitter data as learning data, the performance evaluation may not be applicable to sentence classification because it only focuses on grammatical issues.

Nguyen et al. examined the extraction of events via word2vec and Recurrent Neural Network (RNN) [41]. They termed the most definite word for expressing an event as the event trigger and termed temporary expressions related to the event as the event argument. Word embedding and RNN were used to predict the trigger and argument in an event involving an event trigger and an arbitrary number of event arguments. Random initialization, skip-gram, CBOW and a concatenation-based variant of CBOW (C-CBOW) were used. Specifically, C-CBOW predicted the target word via the concatenation of the vectors surrounding the words. The data set used in the study corresponded to English Gigaword corpus [42]. In the event trigger and argument prediction, C-CBOW exhibited the best performance and Skip-gram exhibited a better performance than that of existing CBOW and Skip-gram. However, CNN and other neural network models were used, and thus, it is necessary to compare CBOW’s and Skip-gram’s performances in CNN-based models.

Additionally, several studies performed in-depth investigations of word2vec. Liu et al. used word2vec to embed a sentence containing citations and analyzed the emotional content by comparing sentences containing positive and negative expressions [19]. Zhang et al. used word2vec and Support Vector Machine (SVM) [43] to classify emotions in comments via extracting features from comments and learning features in comments [20]. In the field of information retrieval, Nalisnick et al. conducted research that searched for related documents by using the similarity between words in the documents [29]. In the field of event detection, Peng et al. proposed a minimally supervised event pipeline (MSEP) that detected events with minimal supervision via word2vec [21]. Specifically, MSEP initially defines the type of event via semantic role labeling, which uses a few examples of data and common nouns and verbs. Subsequently, MSEP uses word2vec to compare the contents of the input document with the contents of the pre-classified event documents and detects events based on their similarity. Verma et al. extracted key sentences in news to detect events from business-related news, analyzed them using word2vec, and then proceeded to classify them based on k-NN [13].

#### CNN

CNN is an artificial neural network that is frequently used for various applications such as image classification, face recognition, and natural language processing [22–24]. In the field of classification, Kim used arbitrarily initialized word vectors and pre-trained word vectors with CNN to classify labeled documents [25]. He analyzed movie reviews [44], Stanford Sentiment Treebank [45], and customer reviews [46] and classified the same. Additionally, the performance of the pre-trained word vector exceeded that of the randomly initialized word vector in the CNN model. Although he indicated that the performance of the proposed CNN model improved the existing classification, he did not confirm that the improvement in performance was due to the training size and the number of learning iterations.

Zhang et al. proposed Multi-Group Norm Constraint CNN (MGNC-CNN) via multiple word embedding to improve the sentence classification performance of CNN [7]. Previous CNN sentences classification techniques used one word embedding while CNN used multiple word embedding to expand the meaning of the incoming word and extract more features. They used word2vec, GloVe [47], and Syntactic word embedding [48] as types of word embedding. Thus, the performance of the MGNC-CNN exceeded those of previous sentence classification algorithms. However, they did not compare the performance of Skip-gram with CBOW in CNN sentence classification because they evaluated the performance by only using CBOW while using word2vec as word embedding.

In the field of event detection, Feng et al. proposed the hybrid neural network (HNN) that combined Bidirectional LSTM (Bi-LSTM) [49] and CNN to create a language-independent neural network [9]. Specifically, Bi-LSTM is a multiple bidirectional recurrent neural network that is capable of simultaneously modeling preceding and following information and word representation. Thus, HNN extracts the features of sentence structures via Bi-LSTM and CNN and determines and classifies event triggers in sentences. The performance of HNN exceeded that of CNN, RNN, LSTM, and Bi-LSTM in the event detection model using English, Chinese, and Spanish. However, only Skip-gram was used, and thus, this did not provide comparison information between Skip-gram and CBOW.

Burel et al. proposed Dual-CNN that added a semantic layer to CNN to solve the event detection problem in a crisis situation from tweets [50]. Each word in the tweet was analyzed and possessed a vector value. Simultaneously, words extracted as named entities in the tweet were entered into the semantic layer with additional vector values. Two word embedding features were extracted via CNN. Dual-CNN extracted more features from the semantic layer and improves accuracy. Thus, they classified the relevance, event type, and information type of the corresponding tweet.

## Classification model

Table 1 shows the hardware specification of the deep learning computer we used. We used Deeplearning4J [4] for the machine learning library, which is the world’s first commercially available open source based library. We built and tested our model through the library. Additionally, we maximized the speed of machine learning via NVIDIA-developed Compute Unified Device Architecture (CUDA) [10]. Specifically, CUDA is a General-Purpose computing Graphics Processing Units technology that enables the use of parallel processing algorithms in the Graphics Processing Unit (GPU). We used CUDA to accelerate the research process via processing CPU-processed operations on the GPU.

**Table 1:** Hardware specification of our deep learning computer.

Item	Detail
OS	Microsoft Windows 10 Pro, 64-bit
CPU	Intel(R) Core(TM) i7-8700
RAM	32.0 GB
Framework	Deeplearning4J
GPU	NVIDIA GeForce GTX 1070
CUDA	9.1

**Fig 3:**
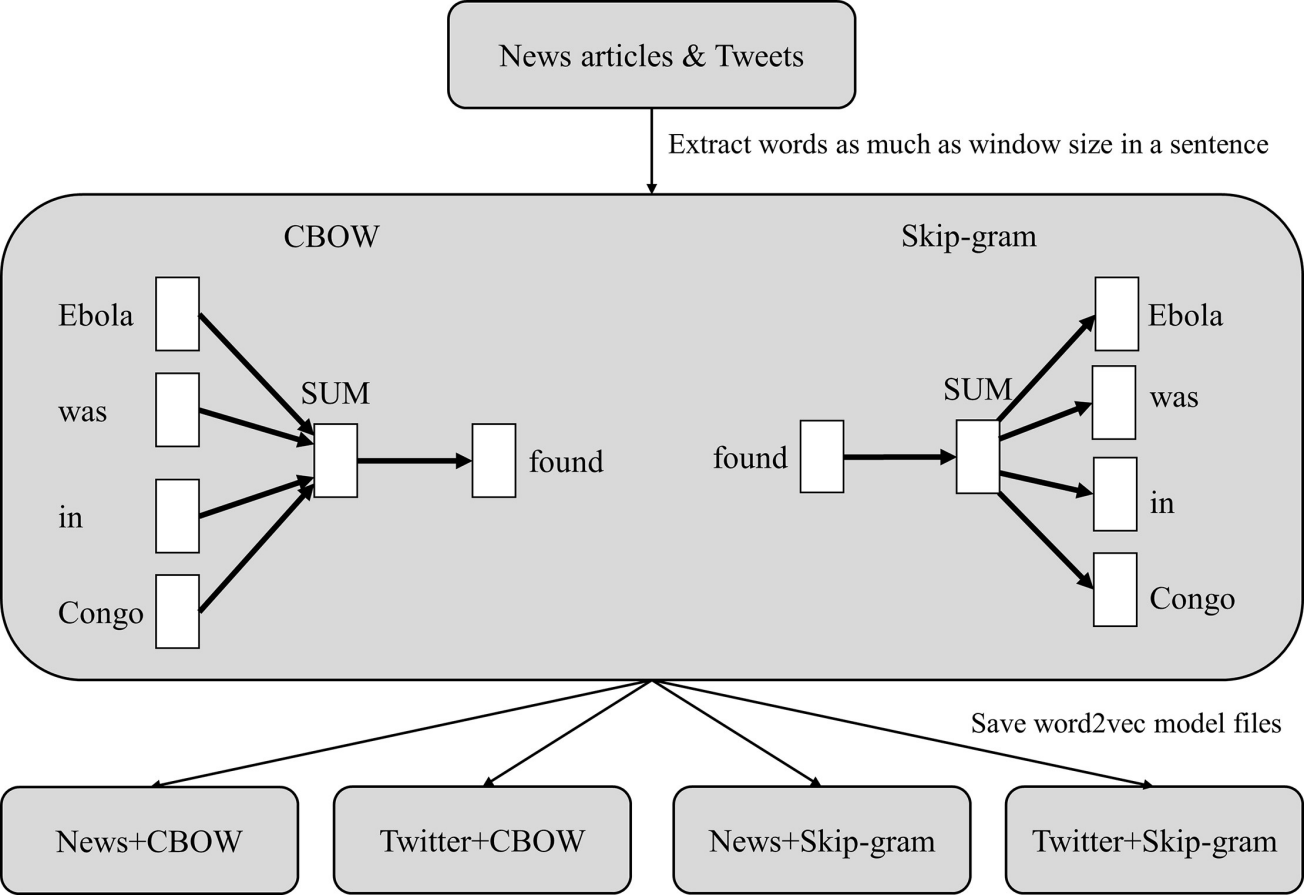
Processing steps of Word2vec.

Fig 3 shows the process for storing the data for word2vec needed by CNN. News articles and tweets were used as training datasets of word2vec CBOW and Skip-gram. Word2vec completed the training and created and saved files containing News and CBOW, Twitter and CBOW, News and Skip-gram, Twitter, and Skip-gram.

Table 2 provides a detailed description of our training datasets for word2vec. We collected news articles and tweets for 62 days from March 1, 2018, to May 1, 2018. We collected approximately 2000 EA news articles per day on average and approximately 4700 EA tweets per day. We collected news articles and tweets via Application Programming Interfaces (APIs) provided by NAVER, which is the most popular portal in Korea, and Twitter. We used fifty-three words related to the disease as keywords for crawling articles and tweets [15, 51].

**Table 2:** Training datasets for word2vec.

Data	Total
Collection Data	March 1, 2018 –May 1, 2018 (62 days)
News articles	122,258
News sequences	2,445,160
News words	160,208,160
Tweets	291,309
Twitter sequences	5,826,160
Twitter words	188,155,940

Table 3 shows keywords used to collect news articles and tweets related to the disease. All data were collected in accordance with the terms of service and privacy of Twitter and NAVER. We removed all unnecessary elements from the collected articles and tweets such as URL and HTML tags. We verified the similarity between articles and tweets and extracted the unique articles and tweets via Sift4 [52] that is a string distance algorithm inspired by Jaro-Winkler [53]. We extracted words from the articles and tweets using Open Korean Text Processor Java (OKTPJ) [54], which is the most widely used Natural Language Processing (NLP) technique in Korea. Subsequently, we performed a tokenization function that delivered a list of tokens consisting of words or tags. Although approximately twice as many significant tweets were collected when compared to news articles, the total number of words extracted through NLP [54] is similar in both categories. This was because Twitter generates more data when compared to news although it communicates through short text and universal words.

**Table 3:** List for disease related keywords.

ENGLISH	KOREAN	ENGLISH	KOREAN
Chicken pox	수두	Fever	발열
Mumps	유행성이하선염	Cough	기침
Thrombocytopenia syndrome	중증열성혈소판감소증후군	Headache	두통
Japanese encephalitis	일본뇌염	Chills	오한
Vibrio vulnificus sepsis	비브리오패혈증	Myalgia	근육통
Legionella’s	레지오넬라증	Abdominal pain	복통
Scrub typhus	쯔쯔가무시증	Diarrhea	설사
Nephrotic syndrome	신증후군출혈열	High fever	고열
Leptospirosis	렙토스피라증	Hemorrhage	출혈
Influenza	인플루엔자	Infection	감염
Scarlet fever	성홍열	Arthralgia	관절통
Hepatitis C	C형간염	Inflammation	염증
CRE	카바페넴내성 장내세균속균종 감염증	Vomiting	구토
Hepatitis A	A형간염	Disease	질병
Syphilis	매독	Illness	질환
Streptococcus pneumoniae	폐렴구균	Syndrome	증후군
Malaria	말라리아	Communicability	전염
MERS	중동 호흡기 증후군	Epidemicity	유행성
Zika virus	지카 바이러스	Symptom	증상
Avian influenza	조류 인플루엔자	Vaccine	백신
Ebola virus	에볼라 바이러스	Incubation period	잠복기
Virus	바이러스	Cold	감기
Detection	검출	Influenza	독감
Prevention	예방	Influenza	인플루엔자
Disinfection	방역	Germ	세균
Definite diagnosis	확진	Bacteria	박테리아
		Occur	발병

Table 4 describes the parameters used in word2vec. We performed several experiments to determine the appropriate parameter values ​​and assigned the values shown in Table 3. We only vectorized words that were used above a minimum frequency threshold. The criterion used specified the use of vectorize words that appeared more than 5 times. If vectorization is performed on words with very low word frequency, performance decreases due to learning of unnecessary words in the document. The iteration referred to the number of iterations of learning. Increases in the number of iterations improved the performance of word2vec because it re-learned the association between words. The learning rate corresponded to an essential parameter for learning, which implies that the initial value of the rate was adjusted while adjusting the weight for learning. In the case of correct learning, the learning rate gradually decreased and, the adjustment of the weight decreased. Layer size denotes the number of dimensions of a word vector. If the layer size is set as extremely small, then it may not be possible to vectorize all the text.

**Table 4:** Parameter of word2vec.

Parameter	Value
Minimum frequency words	5
Iteration	20
Learning rate	0.07
Layer size	1000

**Fig 4:**
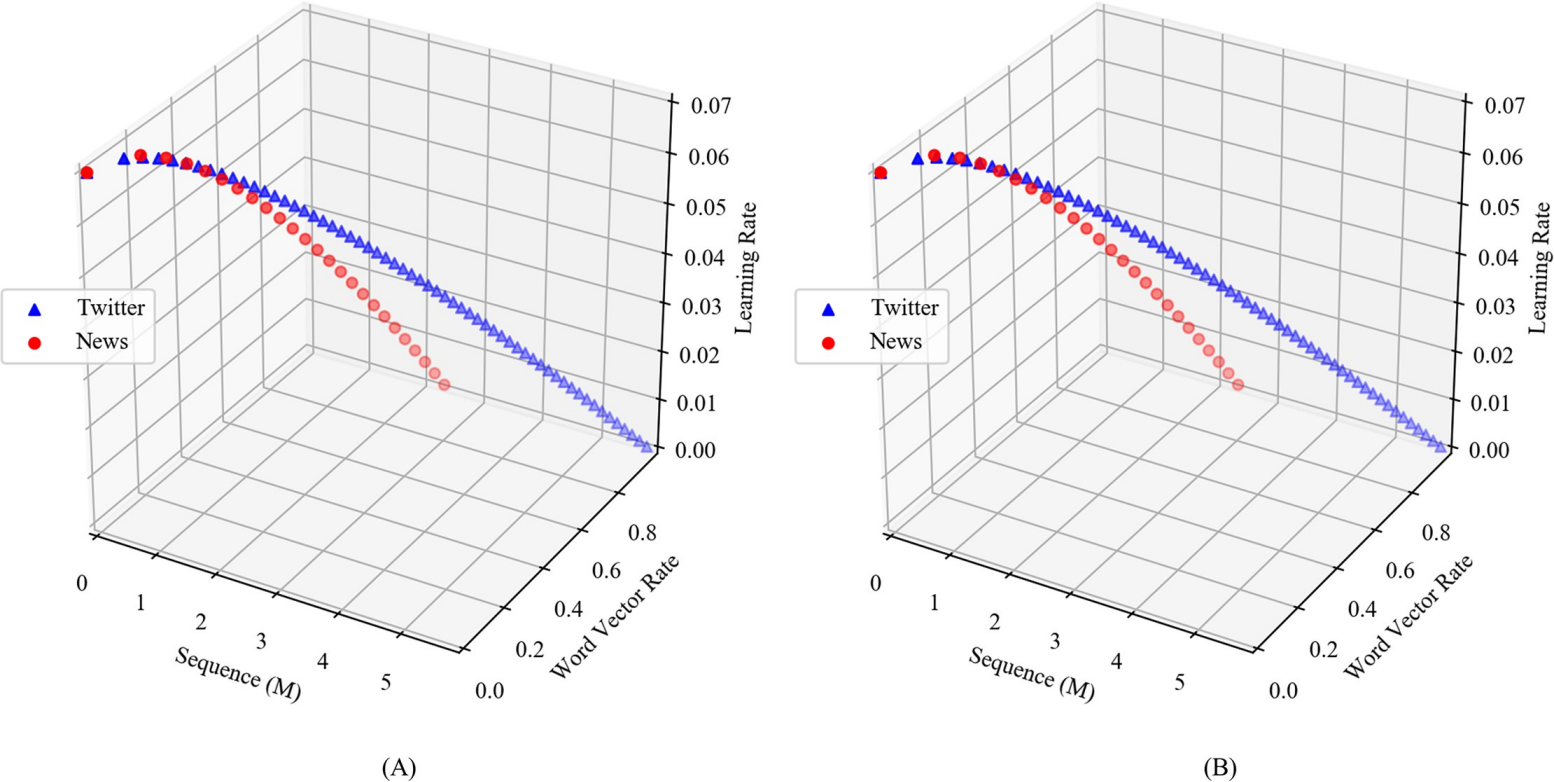
Sequence(M), word vector rate, and learning rate of (a) CBOW and (b) Skip-gram.

Fig 4 shows a three-dimensional graph of (a) CBOW and (b) Skip-gram indicating the relation among sequence, word vector rate, and learning rate. Sequence denotes the number of word groups (window) processed by word2vec and is given in units of millions (M). Word vector rate denotes the rate of vectorization of all words. In the case of news articles, the learning rate of CBOW and Skip-gram decreases sharply when compared to that while learning from tweets. This implies that news articles contain more words in a document when compared to tweets. This is because Twitter limits the length of tweet to 140 characters (or more specifically 280 characters in the USA and 140 characters in Korea, Japan, and China). We did not find any significant differences while comparing CBOW and Skip-gram.

Table 5 shows the data sets and parameters used in the experiment to evaluate the performance of CNN combined with word2vec. We used the cross-validation scheme, in which the training data were different from the test data. The training volume denotes the size of the data used in the learning, and we learned news articles or tweets in various sizes ranging from 1000 to 10000. The training data consists of half of positive and half of negative for news articles and tweets. The test volume denotes the dataset used to evaluate the performance and evaluates performance based on 20000. Test data is not involved in the training, and it is composed of half of positive and half of negative like the training data. Classification denotes whether news articles or tweets were classified as positive or negative. Positive denotes the news articles or tweets we deemed necessary, and negative denotes news articles or tweets we deemed unnecessary.

**Table 5:** Parameter values of CNN.

Parameter	Detail / Value
Training volume	1K – 10K
Test volume	20K
Classification	Positive, Negative
Batch size	500
Epochs	1–200
Feature maps	100

We classified advertising news articles and tweets into negative data and classified news articles and tweets that include disease-related information into positive data. Batch size denotes the number of articles and tweets processed by CNN at a time. Epoch denotes the number of times that CNN learns, and we performed from 1 to 200 iterations of CNN learning. Feature maps denote the depth for each CNN layer.

**Fig 5:**
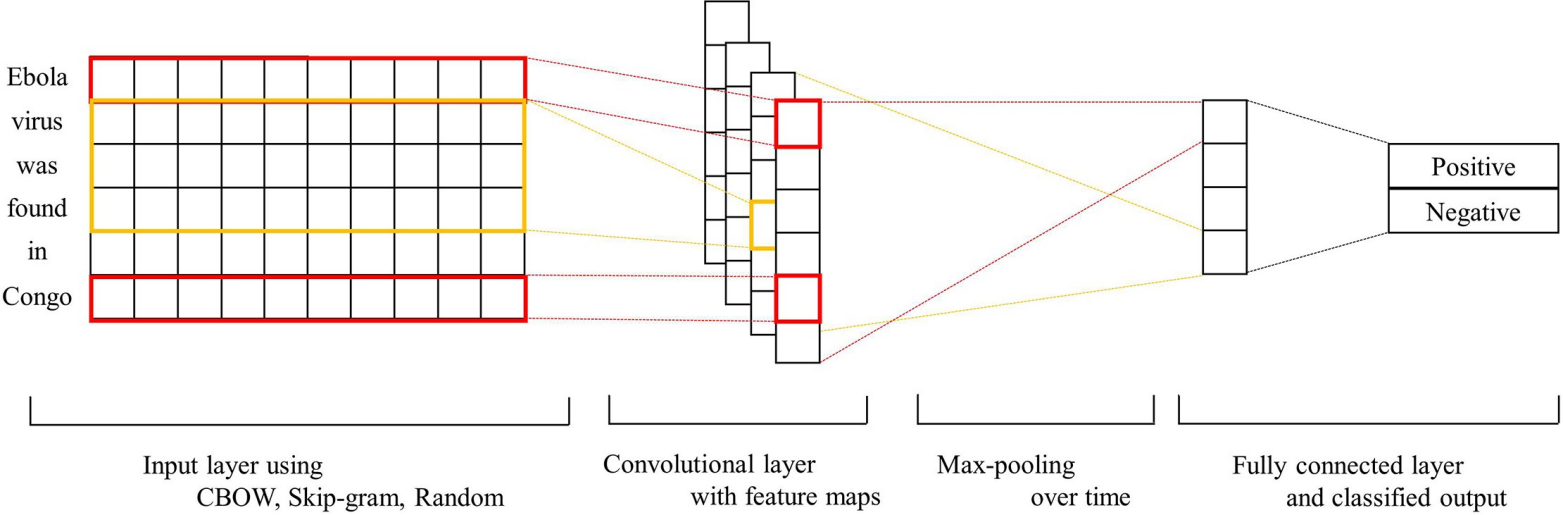
Our CNN architecture with CBOW, Skip-gram, and random learning algorithms.

Fig 5 presents our CNN architecture with CBOW, Skip-gram, and random learning algorithms. Incoming news and Twitter text were tokenized by words, and each token word was assigned a vector value by pre-trained CBOW, Skip-gram, and the random initialization algorithm. Specifically, CNN passed the vectorized value of each tokenized word to the input layer. The input layer extracted features in neighboring words within a specific window size. The process was termed as convolutional operation, and the features extracted through the convolutional operation were used to create a feature map. Subsequently, CNN performed a max-pooling over time operation [24] that extracted the largest value from the extracted features. The process enabled CNN to extract a feature from the article and tweet. The process was repeated by CNN via changing the window size to extract several features. The extracted features were delivered to the fully connected layer, and the dropout and softmax functions were executed. Based on the values, the output layer classified the input text into positive text and negative text. In this process, CBOW, Skip-gram, and the random vector extract different vector values for the same data, so the feature values extracted by the convolutional operation are different. Since the feature values extracted from the same news articles and tweets vary according to used word embedding algorithms, news articles and tweets that are classified as positive text by a word embedding model can be classified as negative text by other word embedding models. We performed CNN classifications with three word embedding model on the same news articles and tweets to find the appropriate word embedding model with high accuracy for news articles and tweets.

## Experiments

In this section, we explore the performances of CNN models with three word embedding, CBOW, Skip-gram, and the random vector for news articles and tweets. We used accuracy, recall, precision, and F1 score[55] as performance metrics. The expressions are as follows:

$$Accuracy=\frac{TP+TN}{TP+TN+FP+FN},$$ (1)

$$Recall=\frac{TP}{TP+FN},$$ (2)

$$Precision=\frac{TP}{TP+FP},$$ (3)

$$F1\;score=2*\frac{Precision*Recall}{Precision+Recall},$$ (4)

where True Positive (TP) denotes the number of real positives among the predicted positives, and True Negative (TN) denotes the number of real negatives of the predicted negatives. Similarly, False Negative (FN) denotes the number of real positives among predicted negatives, and False Positive (FP) denotes the number of real negatives among predicted positives. Therefore, accuracy denotes the proportion of documents classified correctly by CNN among all documents, and recall denotes the proportion of documents that are classified as positive by CNN among all real positive documents. Precision denotes the percentage of documents that are real positive among documents classified as positive by CNN, and the F1 score denotes the average of the weighted recall and precision scores [56].

### News articles

**Fig 6:**
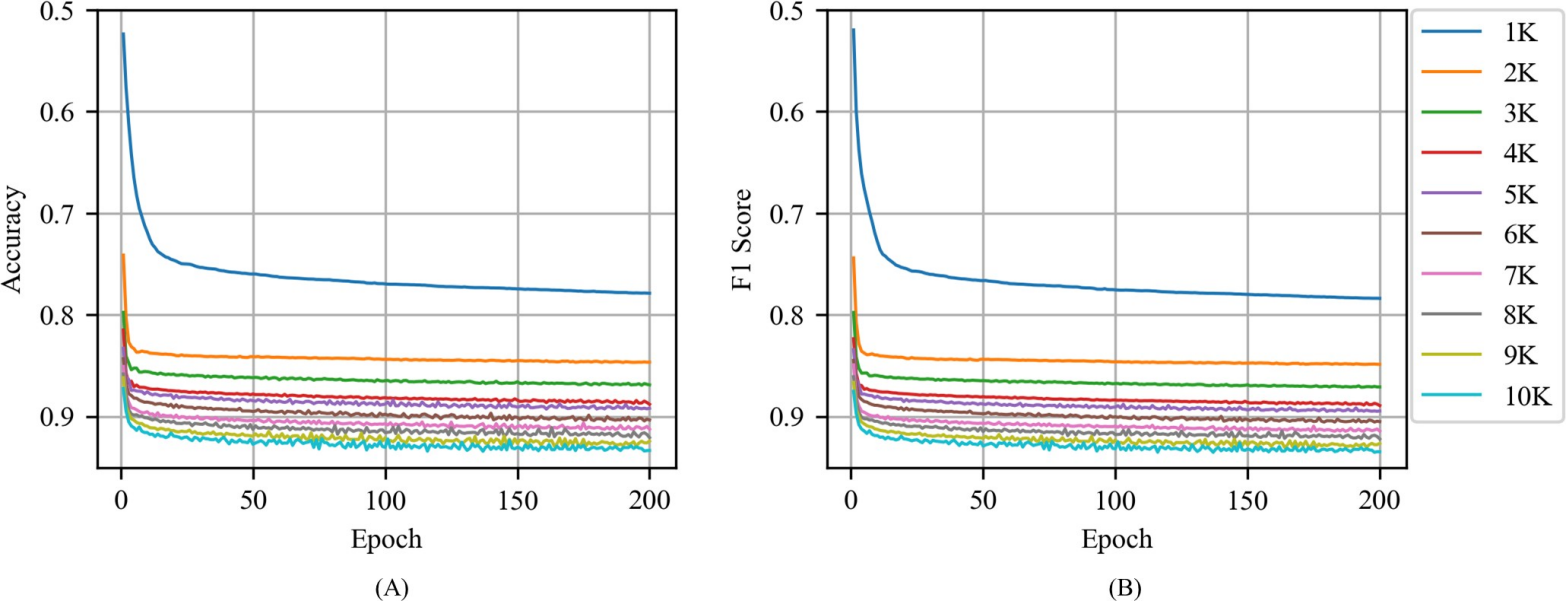
(A) Accuracy and (B) F1 score of CNN with CBOW as a function of epoch for various training volumes in news articles.

Fig 6 shows the accuracy and F1 score of CNN with CBOW as a function of epoch for various training volumes in news articles. The accuracy and F1 score increased when training volume and epoch increased. The training volume exhibited the greatest performance improvement from 1000 to 2000, and the accuracy and F1 score corresponded to 0.85 when the test data set was set at 20000 and the training volume corresponded to 3000. Similarly, the performance of the epoch increased significantly when the epoch increased to 10. Subsequently, it exhibited a fine performance improvement and stable performance.

**Fig 7:**
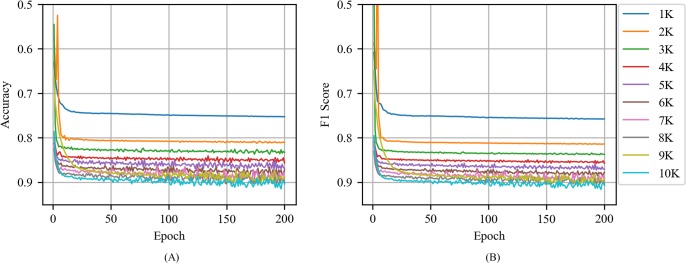
(A) Accuracy and (B) F1 score of CNN with Skip-gram as a function of epoch for various training volumes in news articles.

Fig 7 shows the accuracy and F1 score of CNN with Skip-gram as a function of epoch for various training volume in news articles. A comparison of the CNN with CBOW indicated that the CNN with Skip-gram exhibited a slightly lower performance when compared to CNN with CBOW. Training volumes exceeding 5000 were required to exceed an accuracy and F1 score of 0.85 in CNN with Skip-gram. When the training volume and epoch increased, the accuracy and F1 score values were unstable.

**Fig 8:**
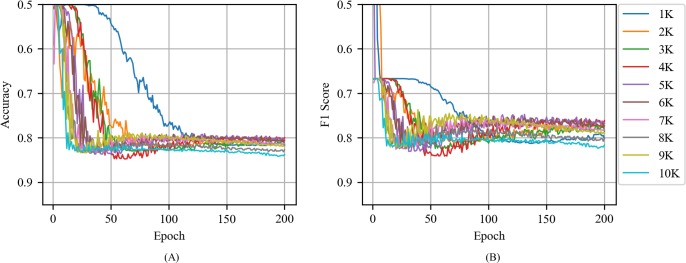
(A) Accuracy and (B) F1 score of CNN with the random vector as a function of epoch for various training volumes in news articles.

Fig 8 shows the accuracy and F1 score of CNN with the random vector as a function of epoch for various training volumes in news articles. Specifically, CNN with the random vector exhibited an unstable performance when compared to the CNNs with word2vec and exhibited a decline in performance when the epoch increased above 30.

### Tweets

**Fig 9:**
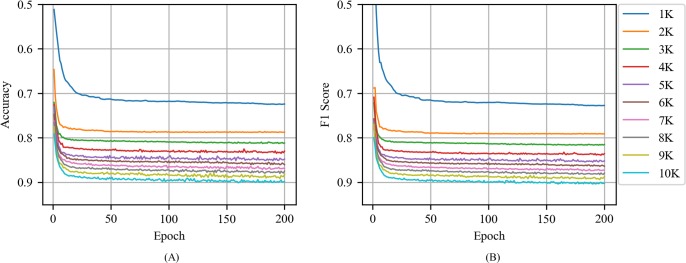
(A) Accuracy and (B) F1 score of CNN with CBOW as a function of epoch for various training volumes in tweets.

Fig 9 shows the accuracy and F1 score of CNN with CBOW as a function of epoch for various training volumes in tweets. Similarities between the CNN with CBOW in news articles are that the accuracy and F1 score increase with increases in the training volume and epoch and that the performance increase is higher when the training volume and epoch value are low. However, the performance of CNN with CBOW in tweets was lower than that of CNN with CBOW in news articles. The accuracy and F1 score of the CNN with CBOW in news articles exceeded 0.85 at the training volume of 3000 and the accuracy and F1 score of the CNN with CBOW in tweets exceeded 0.85 at the training volume of 6000.

**Fig 10:**
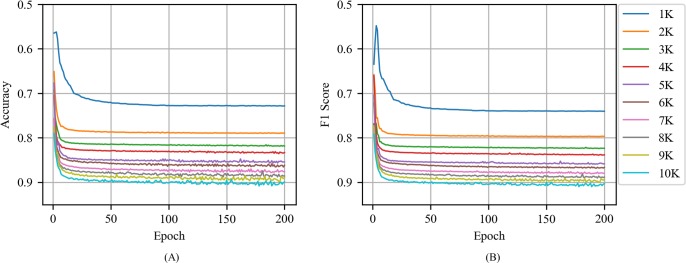
(A) Accuracy and (B) F1 score of CNN with Skip-gram as a function of epoch for various training volumes in tweets.

Fig 10 shows the accuracy and F1 score of CNN with Skip-gram as a function of epoch for various training volumes in tweets. When compared with the CNN with Skip-gram in news articles, the most significant difference was that the accuracy and F1 score values ​​fluctuate unstably when the training volume and epoch increases. Additionally, in contrast to the CNN with Skip-gram in news article, the CNN with Skip-gram in tweets did not exhibit a significant difference from the CNN with CBOW in the same tweet. For news articles, when the training volume size corresponded to 3000 in the CNN with CBOW and 5000 in the CNN with Skip-gram, the accuracy and F1 score values exceeded 0.85. Conversely, for the tweets, both CNN with CBOW and CNN with Skip-gram exhibited an accuracy and F1 score corresponding to 0.85 at a training volume of 6000, and the performance of the CNN with CBOW was similar to that of the CNN with Skip-gram.

**Fig 11:**
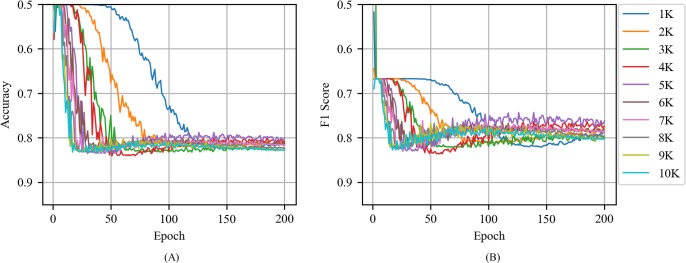
(A) Accuracy and (B) F1 score of CNN with the random vector as a function of epoch for various training volumes in tweets.

Fig 11 shows the accuracy and F1 score of CNN with the random vector as a function of the training volume in tweets. When compared to CNN with the random vector in news article, CNN with the random vector in tweets was less stable and required more epochs to reach its maximum performance. For example, when the training volume corresponded to 2000, the CNN with the random vector in news article exhibited an accuracy of 0.7 or more when the epoch corresponded to 42 although the CNN with the random vector in tweets exhibited an accuracy of 0.7 or more when the epoch corresponded to 56. However, CNN with the random vector in news article and CNN with the random vector in tweets did not exhibit any significant difference in terms of the maximum values of accuracy and F1 score.

## Discussion

**Fig 12:**
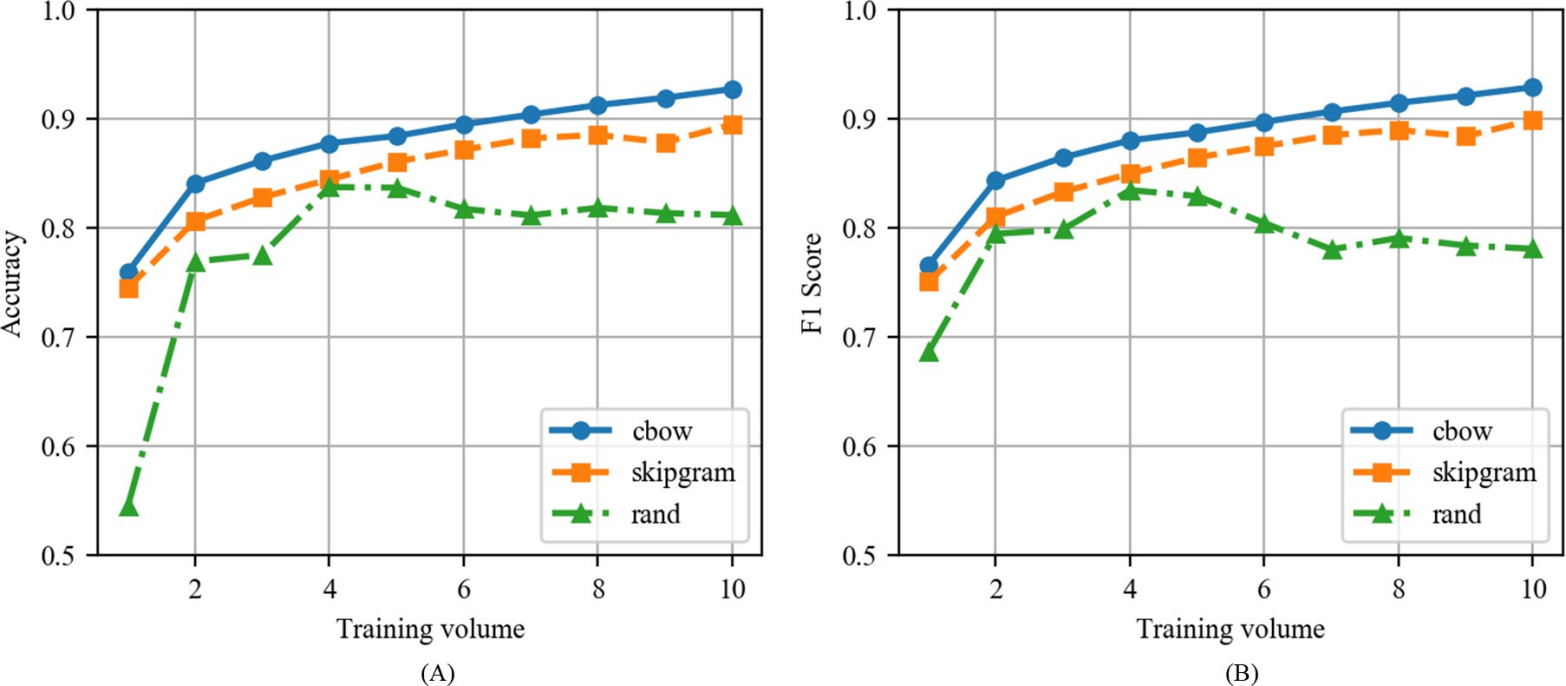
(A) Accuracy and (B) F1 score of CNN with CBOW, CNN with Skip-gram, and CNN with the random vector as a function of training volume when the epoch is fixed to 100 in news articles.

This section analyzes the performance of CNN with CBOW, CNN with Skip-gram, and CNN with the random vector models based on the experiments in Section IV. Fig 12 compares CNN with CBOW, CNN with Skip-gram, and CNN with the random vector as a function of training volume when the epoch is fixed as 100 in news articles. In the same epoch, CNN with CBOW exhibited the highest performance at all training volumes. The CNN with CBOW exhibited values corresponding to 0.9341 and 0.9351, the CNN with Skip-gram exhibited values corresponding to 0.9147 and 0.9161, and the CNN with the random vector exhibited values corresponding to 0.8475 and 0.8409 as the maximum values of accuracy and F1 score. The CNN with CBOW and CNN with Skip-gram exhibited the highest performance at the training volume of 10 although the CNN with the random vector exhibited the highest performance at training volume 4 and subsequently decreased.

**Fig 13:**
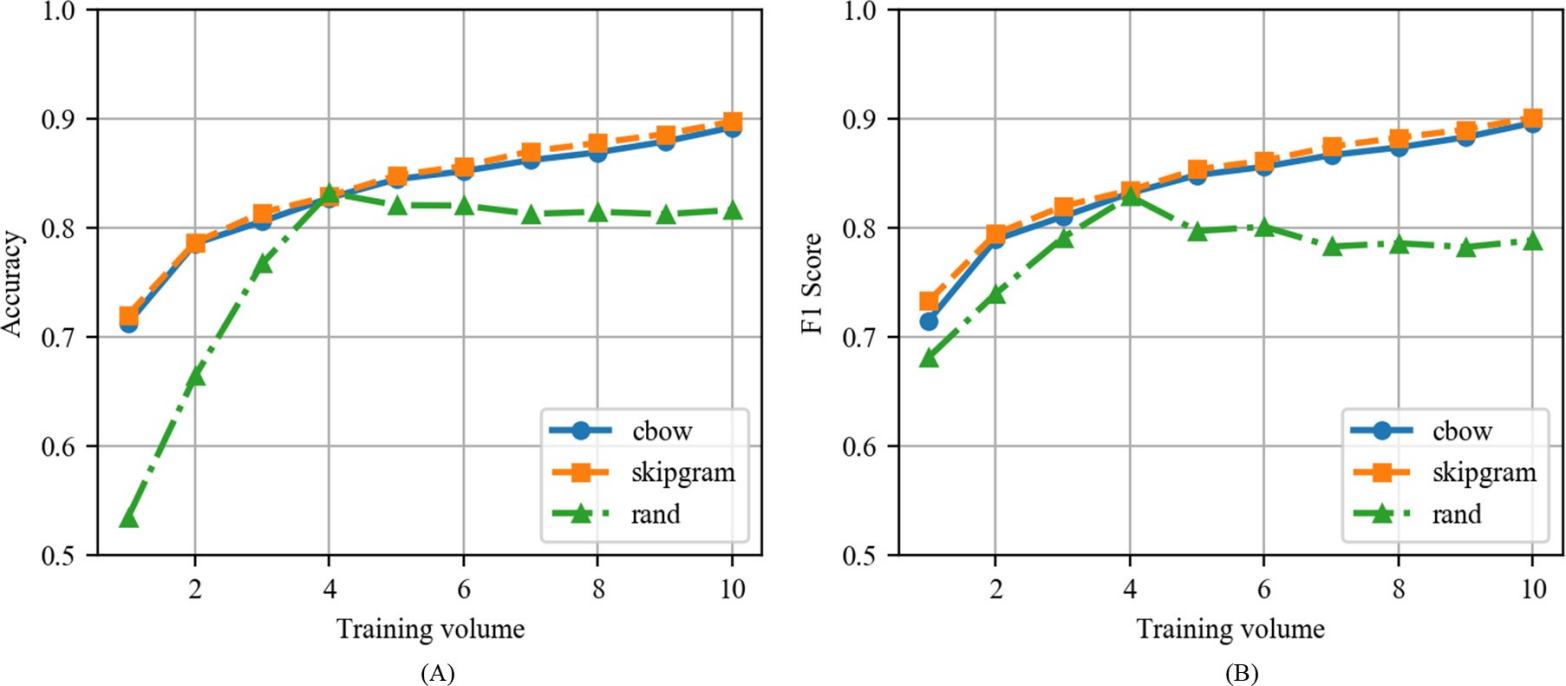
(A) Accuracy and (B) F1 score of CNN with CBOW, CNN with Skip-gram, and CNN with the random vector as a function of training volume when the epoch is fixed to 100 in tweets.

Fig 13 shows the accuracy and F1 score of CNN with CBOW, CNN with Skip-gram, and CNN with the random vector as a function of training volume when the epoch is fixed to 100 in tweets. The performance of CNN with the random vector was similar to its performance when applied to news articles. However, the performances of CNNs with CBOW and Skip-gram were slightly lower when compared to that when applied to news articles. Significant differences in performance between CNN with CBOW and CNN with Skip-gram were not observed when applied to tweets.

Table 6 shows the highest accuracy and F1 score for each algorithm, and the epoch value and training volume value used when the corresponding value was output. The CNN with CBOW model in news articles exhibited the highest overall performance although the CNN with Skip-gram model performed better when compared to the CNN with CBOW algorithm in the tweets. With respect to all models (with the exception of the random algorithm), increases in the training volume and epoch improved the performance. Additionally, the performance significantly reduced when CNN with the random vector without word2vec was used. This indicated that word embedding for learning the relationship between words is an important factor in classification using CNN.

**Table 6:** Experiments analysis.

Model	Accuracy	F1 score	Training Volume	Epoch
News + CBOW	**0.9341**	**0.9351**	10	137
News + Skip-gram	0.9147	0.9161	10	197
News + Random	0.8475	0.8409	4	62
Twitter + CBOW	0.9010	0.9037	10	160
Twitter + Skip-gram	**0.9081**	**0.9097**	10	178
Twitter + Random	0.8403	0.8360	4	60

This paper has following advantages. It found that the CNN classification model with word2vec such as CBOW and Skip-gram algorithms outperformed the CNN classification model with the random vector. It means that the proposed CNN classification model used with word2vec is better than the CNN classification model without word2vec. In the case of news articles, the CNN classification model with CBOW had higher performance, but the CNN classification model with Skip-gram showed higher performance for tweets. It means that the appropriate word embedding algorithm for performance enhancement may vary depending on the data type. Our paper has following disadvantages. We need to evaluate performances of other word embedding algorithms such as GloVe [47], Fasttext [57]. We also need to consider other types of data such as customer review and movie review.

## Conclusion

In the study, we evaluated the use of word2vec in classification models via CNN based on news articles and tweets. We examined the effect of using word2vec on the results and compared the performance of two of word2vec’s learning algorithms, namely CBOW and Skip-gram. We observed that the use of word2vec that learned semantic relations among words significantly improved the performance of classification models. The results confirmed that the CBOW algorithm performed better when used on news articles and that the Skip-gram algorithm exhibited a better performance when used on tweets. This implied that the use of different algorithms based on the type of data to be analyzed can yield a better performance. All models exhibited better performance on news articles when compared to that on tweets. News articles typically exhibit a more uniform format when compared to tweets, and thus, CNN models could extract features and perform faster accurate classification when formatted data was entered into the CNN-based classification model. Thus, we examined the impact of well-learned word embedding on news articles and tweets classification via CNN and presented appropriate word embedding models based on the type of data

## Future works

We have three ongoing future research works. Firstly, we have to consider various word embedding techniques. We considered CBOW and Skip-gram of word2vec as our word embedding algorithm because word2vec has widely considered to one of the best word embedding algorithms [58–60]. Although we found appropriate word embedding models for news articles and tweets, our experimental results could be applied only to Word2vec's CBOW and Skip-gram. We must compare word2vec with other word embedding techniques such as GloVe [47] and Fasttext [57]. Secondly, we have to consider various web big dataset. We considered news articles and tweets as datasets because they were considered to ones of the most representative web big data. We must consider various web big data such as customer review and movie review. The length limit of tweets is relatively short as 280 characters per each tweet and common length of tweet is only 33 characters. We also must apply our proposed technique on longer review data than tweets. Thirdly, we can exploit our proposed CNN classification model to predict the outbreak of infectious disease. Disease-related news articles and tweets must correlate with the outbreak of infectious diseases and are used to predict it [61–63]. However, other purpose texts such as advertisement may deteriorate the accuracy of predictions. We will use our proposed CNN classification model to improve the accuracy of the previous disease prediction model.
